# The Crystal Structure of a *Streptomyces thermoviolaceus* Thermophilic Chitinase Known for Its Refolding Efficiency

**DOI:** 10.3390/ijms21082892

**Published:** 2020-04-21

**Authors:** Piotr H. Malecki, Magdalena Bejger, Wojciech Rypniewski, Constantinos E. Vorgias

**Affiliations:** 1 Institute of Bioorganic Chemistry, Polish Academy of Sciences, Noskowskiego 12/14, 61-704 Poznan, Poland; piciumalecki@gmail.com (P.H.M.); bejger@ibch.poznan.pl (M.B.); 2 Department of Biology, Section of Biochemistry and Molecular Biology, National and Kapodistrian University of Athens, 15701 Zografou, Greece; cvorgias@biol.uoa.gr

**Keywords:** X-ray crystallography, structure and function relationship, glycoside hydrolase, chitinase, thermostability, TIM-barrel, α+β-domain, *Streptomyces thermoviolaceus*

## Abstract

Analyzing the structure of proteins from extremophiles is a promising way to study the rules governing the protein structure, because such proteins are results of structural and functional optimization under well-defined conditions. Studying the structure of chitinases addresses an interesting aspect of enzymology, because chitin, while being the world’s second most abundant biopolymer, is also a recalcitrant substrate. The crystal structure of a thermostable chitinase from *Streptomyces thermoviolaceus* (*St*Chi40) has been solved revealing a β/α-barrel (TIM-barrel) fold with an α+β insertion domain. This is the first chitinase structure of the multi-chitinase system of *S. thermoviolaceus*. The protein is also known to refold efficiently after thermal or chemical denaturation. *St*Chi40 is structurally close to the catalytic domain of psychrophilic chitinase B from *Arthrobacter* TAD20. Differences are noted in comparison to the previously examined chitinases, particularly in the substrate-binding cleft. A comparison of the thermophilic enzyme with its psychrophilic homologue revealed structural features that could be attributed to *St*Chi40’s thermal stability: compactness of the structure with trimmed surface loops and unique disulfide bridges, one of which is additionally stabilized by S–π interactions with aromatic rings. Uncharacteristically for thermophilic proteins, *St*Chi40 has fewer salt bridges than its mesophilic and psychrophilic homologues.

## 1. Introduction

Chitin, known as the “marine cellulose”, is an insoluble linear β-1,4-linked polymer of N-acetyl-β-glucosamine. It is degraded by chitinolytic enzymes and it has been gradually capturing interest of the biotechnological community. Chitinolytic enzymes, chitinases (EC 3.2.1.14), mainly comprise the GH18 and GH19 families of the glycoside hydrolase (GH) superfamily and have been found in a wide range of organisms, such as fungi, crustaceans, and insects, which contain chitin in their tissues, as well as organisms that do not contain chitin, such as archaea, bacteria, viruses, higher plants, animals, and humans [1,2,3,4]. Classification of chitinases is based on the primary structure comparisons, but it is well established that chitinases belonging to the same family share the main properties, such as the fold of the catalytic domain, substrate specificity, stereochemistry of the reaction, as well as the catalytic mechanism [5,6]. The most studied microbial chitinases are from the *Serratia marcescens* enterobacteria noted for their simple but efficient chitin degradation system [7,8].

Among the *Actinomyces*, the Gram-positive soil bacteria of the *Streptomyces* genus are particularly efficient in breaking down chitin, which they use as a source of carbon and nitrogen. [9,10]. In the past years, several reports have described chitinolytic enzymes, while the corresponding genes have been isolated and characterized. At the level of gene regulation, several gene control elements of the chitinolytic system have been studied in the *Streptomyces* [11,12,13]. In thermostable bacterium *Streptomyces thermoviolaceus* OPC-520, the chitinolytic system consists of four chitinase genes (*chi*40, *chi*35, *chi*30, and *chi*25) and two N-acetyl-β-glucosaminidase genes (*nag*A and *nag*B). Comparisons of the deduced amino acid sequences of the four chitinase genes revealed that the *St*Chi40 and *St*Chi30 proteins belong to family GH18, while *St*Chi35 and *St*Chi25—to family GH19. *St*Chi40, *St*Chi30, and *St*Chi25 have been determined as active enzymes, while the *chi*35 gene product is inactive against chitin [10]. We found *St*Chi40 to refold completely and repeatedly after thermal unfolding [14]. We considered *St*Chi40 a suitable model protein for studying the molecular basis of thermostability and reversible folding of thermostable enzymes based on the TIM-barrel fold.

Members of the GH18 family display a multi-domain architecture with various arrangements of the domains. The common feature is a catalytic domain with the β/α-barrel fold, whose function is to hydrolyze the β-1,4 linkage between N-acetyl-d-glucosamine residues of chitin. Chitinases of the GH18 family contain a characteristic DxDxE amino acid sequence motif, in which glutamate is the catalytic residue essential for activity. In many chitinases, catalytic domains contain inserted subdomains. However, in only a few cases their function is assigned. For instance, deletion of the α+β insertion in ChiA of *S. marcescens* affected geometry of the catalytic groove. As a result, the specific activity was greatly reduced, the pH optimum was shifted, and the chitinolytic degradation pattern also included NAG [15]. Some chitinolytic enzymes contain specialized carbohydrate-binding modules (CBMs) designed to bind chitin, known as chitin-binding domains (ChBD). They are found in plant, fungal, and bacterial proteins and are structurally diverse. In addition, many chitinases contain fibronectin type III (FnIII) or immunoglobulin-like (Ig-like) domains. One or more of them can be found between the catalytic domain and the ChBD. They are postulated to serve as spacers to adjust the position of the catalytic domain on the surface of chitin. Unfortunately, it is difficult to elucidate the structural basis of this interaction. In few cases, only the interaction of NAG oligomers docked in the catalytic groove has been structurally studied in atomic resolution [16,17,18].

In this work, we present the crystal structure of *St*Chi40, which we have examined and compared with related chitinases, including a cold-adapted homologue. We have noted similarities and distinguishing features that could contribute to its thermostability.

## 2. Results

### 2.1. The Overall Structure of StChi40

**Figure 1:**
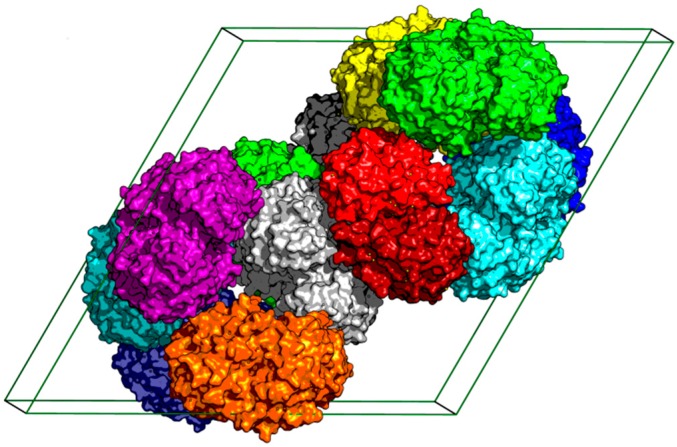
Crystal packing of *St*Chi40 molecules. The two molecules in each asymmetric unit are shown in the same color. In the pair of molecules colored red, the two monomers are distinguished by different shades. The unit cell is shown as a black box.

There are two *St*Chi40 molecules in the asymmetric unit, related by a non-crystallographic two-fold axis; the molecules contact each other via their nearly flat surfaces that contain the substrate-binding groove and the catalytic site. The pairs of *St*Chi40 molecules are packed around the 6_1_ axis (Figure 1).

**Figure 2:**
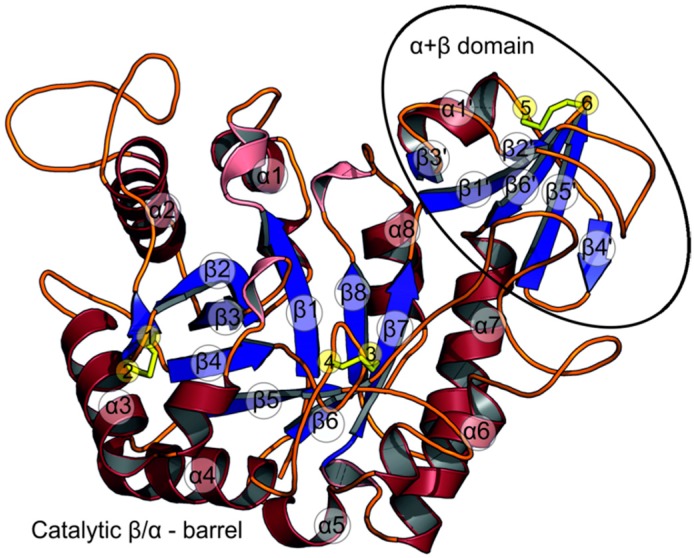
Cartoon plot of *St*Chi40. The two protein domains are labelled. The secondary structure elements—α-helices (red), β-sheets (blue), 3_10_-helices (pink)—of the catalytic domain are labelled according to the convention for β/α-barrels. The α+β insertion domain is marked by an oval. Cysteine residues forming disulfide bridges are labelled (yellow sticks) and numbered: 1—Cys86, 2—Cys162, 3—Cys188, 4—Cys192, 5—Cys343, 6—Cys357.

The model of molecule A consists of 369 amino acid residues (40–408), while the model of molecule B consists of 370 amino acid residues (39–408). Both are very similar, with the root-mean-square (r.m.s.) deviation of 0.23 Å for the main chain atoms. The protein consists of the N-terminal catalytic β/α-barrel domain (classed as GH18) and an α+β insertion subdomain that is mainly composed of five antiparallel β-strands and an α-helix. The insertion domain is located between strand β7 and helix α7 of the main domain and also comprises a part of the loop between strand β6 and helix α6 (Figure 2).

The structures most similar to *St*Chi40, with amino acid sequence identity within the *St*Chi40 domains, are the catalytic domain of psychrophilic chitinase B from *Arthrobacter* TAD20 (37.1% identity, PDB code 1kfw), the catalytic domain of chitinase A1 from *Bacillus circulans* WL-12 (34.3%, PDB code 1itx), chitinase CrChi1 from nematophagous fungus *Clonostachys rosea* (33.3%, PDB code 3g6l) [19], ChiA from *Serratia marcescens* (32.0%, PDB code 1ctn) [20], chitinase B from fungus *Aspergillus niger* (33.7%, PDB code 6igy), chitinase-h from insect *Ostrinia furnacalis* (30.5%, PDB code 5gpr) [21], ChiA74 from *Bacillus thuringiensis* (30.3%, PDB code 6bt9) [22], and chitinase from *Vibrio harveyi* (30.8%, PDB code 3b8s) [17]. Also, among similar chitinases there is another thermophilic chitinase from fungus *Rhizomucor miehei* (29.2%, PDB code 5xwq).

**Figure 3:**
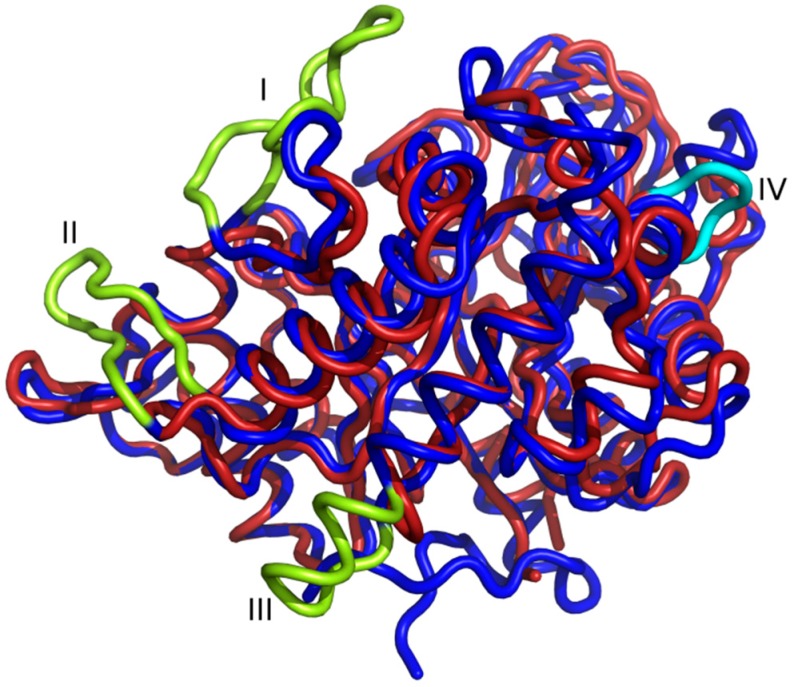
Superposition of *St*Chi40 (red) with psychrophilic chitinase B from *Arthrobacter* (blue). Loops absent in *St*Chi40 are shown (lime): between β2 and α2 (I), between α3 and β4 (II) and between α4 and β5 (III); the loop absent in chitinase B between β5 and α5 is colored cyan.

A comparison of *St*Chi40 and the chitinase from psychrophile *Arthrobacter* reveals truncations in the thermophilic *St*Chi40 with respect to the cold-adapted protein, in particular on the substrate-binding side of the β/α-barrel, where two adjacent extended loops are trimmed in *St*Chi40: between β2 and α2 and between α3 and β4. Another truncation in *St*Chi40 is observed between α4 and β5, where the *Arthrobacter* chitinase has a surface loop (Figure 3). The same three loops are also shortened in a chitinase from thermophilic *R. miehei*. Contrary to the trend of loop shortening, a small insertion has taken place in *St*Chi40 between β5 and α5, in comparison to the psychrophilic protein.

### 2.2. Substrate-Binding Groove

**Figure 4:**
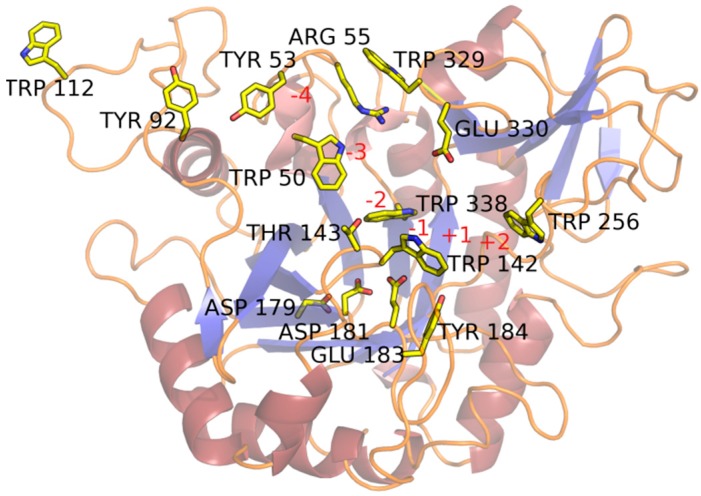
The substrate-binding site of *St*Chi40. Residues lining the substrate-binding cleft are shown and discussed in the text. Subsites are labelled red. Solvent molecules are not shown.

**Figure 5:**
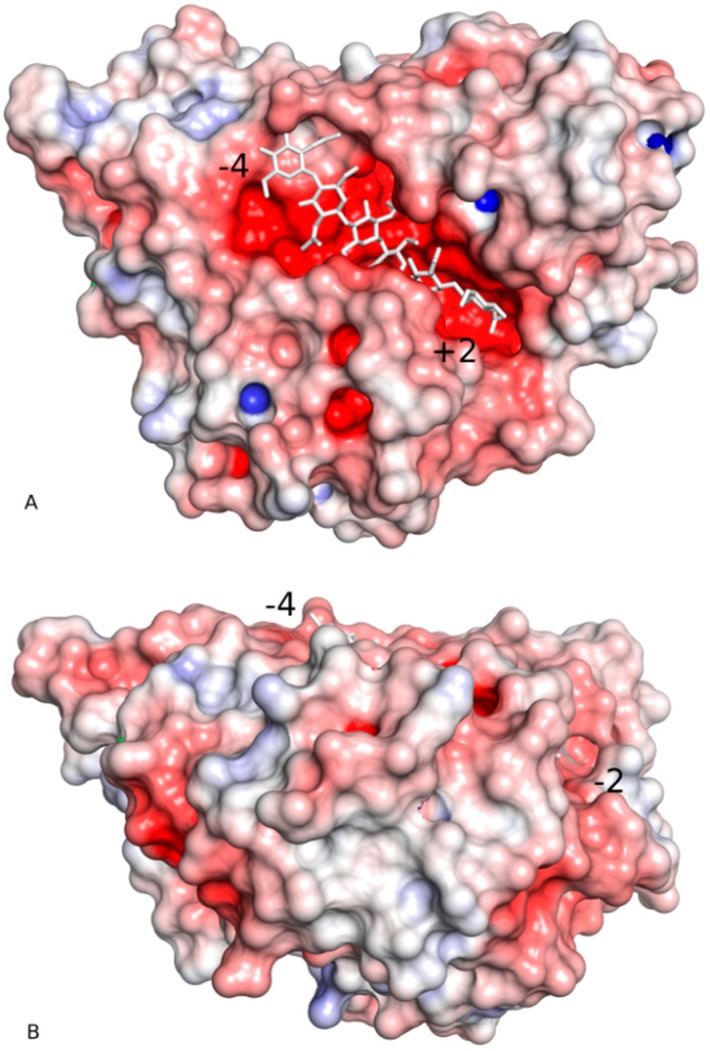
Two perpendicular views of the electrostatic surface representation of *St*Chi40 showing the substrate-binding groove (**A**) and the side view of the flat surface that contains the groove (**B**). Panel A shows the molecule in the same orientation as in Figure 2 and Figure 4. The electronegative (red) character of the binding site is clearly visible. The insertion domain is at the top-right side of panel A. The substrate (white sticks) is modelled by superposing chitinase A from *Vibrio harveyi* complexed with NAG_6_ (PDB code 3b9a) onto *St*Chi40 [17]. Terminal substrate-binding subsites are labelled.

The substrate-binding groove is located at carboxyl ends of the β-strands that constitute the β/α-barrel, similar to other enzymes with the TIM-barrel architecture. Residues belonging to the loops that connect the β-strand with α-helixes make up the substrate-binding cleft. The groove is deep and clearly defined (Figure 4 and Figure 5A).

The surface of the protein around the substrate-binding groove is remarkably flat, suggesting a close interaction with the chitin’s facet (Figure 5B). The insertion domain, in addition to forming part of the substrate-binding cleft, contributes to this planar surface (Figure 4 and Figure 5A). *St*Chi40 lacks a chitin-binding domain which extends from the “–“ site and is observed in ChiA from *S. marcescens*, chitinase-h from insect *O. furnacalis,* and chitinase from *V. harveyi*.

The groove in the *St*Chi40 crystal structure is occupied by water molecules and a molecule of malonate ion wedged in the slot between the rings of Trp142 and Trp256 in each of the two protein molecules present in the asymmetric unit (Figure 4). Although there is no substrate in the *St*Chi40 crystal structure, the residues lying along the substrate-binding groove and the active site residues have been identified based on a comparison with an inactive mutant of ChiA from *S. marcescens* complexed with NAG_8_ [16] (PDB code 1ehn) or ChiA from *V. harveyi* complexed with NAG_6_ (PDB code 3b9a) [17]. The residues that make H-bonding and stacking interactions with the ligand are highly conserved among the related chitinases mentioned above. In *St*Chi40, the catalytic residue is Glu183, a part of the DxDxE signature. Its side chain is directed towards the ligand-binding groove near the scissile bond. The second D, Asp181, is conserved, and it has been observed to rotate between the other two residues of the conserved motif, as it stabilizes the glutamate in an active form and contributes to an essential distortion of the N-acetyl group of the substrate’s −1 sugar [23]. According to the ChiA structure in complex with a poly-NAG, Trp142 and Trp256 form a cleft for binding the ligand. Tyr184, which substitutes Phe316 from ChiA, has an additional hydroxyl group to bind the ligand at the +1 subsite (Figure 4). What appears to be a continuous helix α2 is composed of two segments interrupted by a long loop (96-117) (Figure 2) which extends the substrate-binding patch on the “–“ subsite, adding to it solvent-exposed Trp112 at the protein’s fringe (Figure 4).

The “–“ side of the substrate-binding groove is lined with aromatic residues conserved in all the similar chitinases: Trp338 and Trp50 followed by Tyr53 and Tyr92. The last aromatic residue on this patch is Trp112. There are several other residues in positions equivalent to those interacting with ligand in ChiA: Glu330, Trp142, Thr143, Trp329, Arg55 (Figure 4). In *St*Chi40, Asp54 that points towards the substrate-binding groove is substituted by Gly in most of the other structures but not in insect chitinase-h in which Pro residue is observed.

### 2.3. Disulfide Bridges

**Figure 6:**
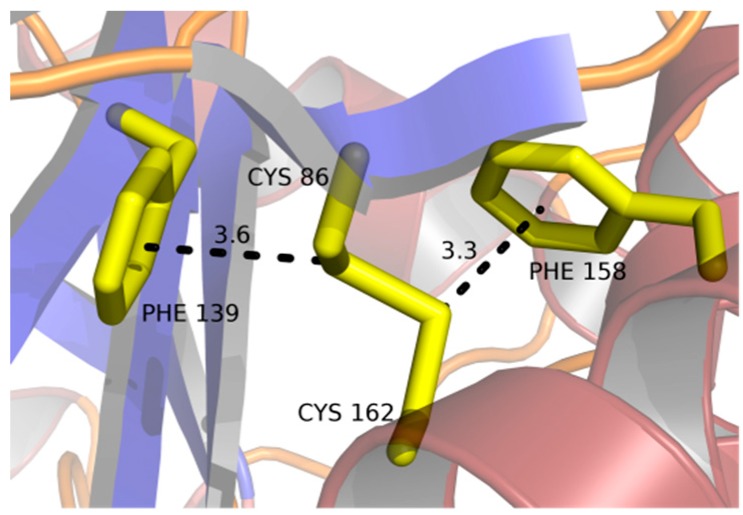
Disulfide bridge between Cys86 and Cys162 which includes S–π interactions with aromatic rings. Distances are indicated in angstroms.

All the cysteine residues are involved in intra-chain disulfide bridges (Figure 2). The first pair (Cys86 and Cys162) links a short β-strand that follows strand β2 with helix α3 that runs parallel to it, although it is sequentially distant. This bond attaches the N-terminal end of the extended surface loop between β2 and α2 to the body of the β/α-barrel. Both sulfur atoms interact with aromatic rings: S atom of Cys86 at a distance of 3.6–3.9 Å to the centroid of Phe139 and S atom of Cys162 at 3.3–3.5 Å to the centroid of Phe158 (Figure 6). In the chitinase from psychrophilic *Arthrobacter*, a disulfide bridge is present in an equivalent position, but with no aromatic interactions. Additionally, Phe139 in *St*Chi40 is unique among the most similar structures listed above.

The second pair of cysteine residues (Cys188, Cys192) makes a disulfide bond at the base of the β-turn between strand β4 and helix α4 (Figure 2). The cystine is exposed to the solvent. The tip of this β-turn, Leu190, interacts with conserved substrate-binding Trp142. This disulfide bond is unique among the compared chitinases.

The third disulfide bridge (Cys343 and Cys357) is found in the insertion domain (Figure 2). This covalent bond connects the end of helix α1’ with the end of strand β5’. The S atom of Cys343 lies close to the plane of the Trp362 aromatic ring, within 3.3–3.5 Å from the Cδ1 atom. This disulfide bond is also unique among the similar chitinases.

### 2.4. Amino Acid Substitutions and Their Structural Consequences

The features potentially stabilizing the structure have been examined. First, all salt bridges have been identified [24] and analyzed in terms of evolutionary conservation. Among the most similar structures, comparing only superimposable structural elements, there are fewer interactions in *St*Chi40 between positively (His, Lys, Arg) and negatively charged residues (Glu, Asp) than between the comparable meso- and psychrophilic proteins: five salt bridges in *St*Chi40 compared to eight up to thirteen in the related enzymes. However, the number of salt bridges in the other thermophilic chitinase from *R. miehei* is even lower: two (Table 1).

Some residues that are conserved in other proteins are substituted in *St*Chi40 (Table 1). For example, instead of Arg, Lys, or Gln commonly observed in position 167, a His residue is present in *St*Chi40 and its nitrogen atoms interact with Oδ1 of Asp172 and Oε1 of Glu214. It is worth mentioning that Glu214 taking part in this interaction is also unique, because an aromatic residue is usually placed in this position. The other thermophilic chitinase, from *R. miehei*, has Thr218 in this place. Pro155 and Pro264 observed in *St*Chi40 are unique and substitute Arg and His, respectively, commonly observed in mesophilic and psychrophilic chitinases. Interestingly, the other thermophilic chitinase has hydrophobic residues (Phe and Ala) in these two positions.

## 3. Discussion

Unlike many chitinases, which often contain accessory domains in addition to the catalytic domain, *St*Chi40 has a relatively simple structure consisting of a single TIM-barrel β/α domain with an α+β insertion subdomain that augments the substrate-binding site. It was found to fold reversibly with high efficiency, which made it an interesting model to study the folding/unfolding mechanism of a thermostable TIM-barrel [14]. Crystallization of *St*Chi40 was achieved after 20 years of attempts by several research groups, which is somewhat surprising given the protein’s stability, and the crystal structure was solved using a single available crystal.

*St*Chi40 shares the features characteristic of GH18, having the β/α-barrel fold of the catalytic domain and conserved sequence motif DxDxE. Conservation of the residues that take part in catalysis indicates that the reaction mechanism of chitin degradation proceeds according to the established scheme for chitinases of this family [25]. *St*Chi40 has a deep substrate-binding groove, similar to that found in ChiA from *S. marcescens* [20]. This can be contrasted with a shallow binding site of *Mm*Chi60, a chitinase from a psychrophilic bacterium [26]. It was argued that shallowness of the binding groove could be a feature in cold-adapted enzymes to increase accessibility of the substrate, whereas in thermophilic and mesophilic enzymes, optimizing substrate accessibility should not be as critical as in psychrophiles.

It has been noted that there are many pathways by which extremophilic enzymes can adjust to their environments and various features have been identified as being significant for adaptation to different temperatures. In the course of evolution, enzymes have adjusted the number, kind, and strength of their internal interactions to optimize the balance between rigidity—in order to be stable—and flexibility, to be active at their environmental temperatures. Loop trimming, disulfide bonds, salt bridges, and proline residues are the features noted to occur in thermostable proteins. Thermophilic *St*Chi40 shares 37% of its amino acids sequence with the catalytic domain of a chitinase from *Arthrobacter* TAD20, its cold-adapted structural counterpart. A comparison of the two structures is interesting with regard to the question of the proteins’ adaptation to different temperatures.

Since thermal stability is achieved by the cumulative effect of multiple small contributions, it is difficult to formulate general rules for such enzymes. However, alignment of *St*Chi40 with psychrophilic ChiB from *Arthrobacter* shows that *St*Chi40 has shorter surface loops (Figure 3). A similar loop arrangement is observed in the chitinase from thermophilic *R. miehei*. Surface minimization could be a factor in achieving thermostability and an almost 100% refolding of *St*Chi40 after thermal denaturation. Mobility is the highest in loops, thus they are the likely initiation points for thermal denaturation. Shortening of loops has been observed in thermophilic proteins, and it was interpreted in terms of restricting conformational freedom of the unfolded state [27]. This should reduce the difference in entropy between the unfolded and folded state and thus make it easier for the protein to refold. Minimizing the ratio of the surface area to volume enhances stability of the protein by reducing the unfavorable surface energy and increasing the interior packing interactions [28]. There is some experimental evidence that trimming surface loops by protein engineering can increase thermostability, although in practice it is difficult to modify proteins in such a way that only one aspect is probed and the structure is not disturbed in some other way [29].

Disulfide bridges are commonly found in extracellular proteins and even in cytosolic proteins in heat-stable organisms, as in the case of the adenylosuccinate lyase from hyperthermophilic *Pyrobaculum aerophilum* [30]. They are considered a significant factor in stabilizing their structures [31]. Studies also point to other stabilizing forces in heat-resistant proteins, such as hydrophobic interactions, ionic interactions, and hydrogen bonds. However, on a one-to-one basis, the strength of covalent bonding exceeds by far the other interactions. In *St*Chi40, all six Cys residues are paired up as disulfide bridges. One of the cystines, made by Cys86 and Cys162, is additionally stabilized by S–π interactions with Phe residues (Figure 6). This kind of interaction has been observed in hydrophobic cores of proteins and is believed to contribute to protein stability [32]. It is strongest when the S atoms lie directly opposite the centroid of the aromatic ring [33], which is exactly what is observed here with both sulfur atoms. A corresponding disulfide bridge in the psychrophilic chitinase B lacks the aromatic interactions. Other comparable chitinases from mesophilic organisms do not share this feature either. The other two disulfide bridges observed in *St*Chi40 are unique among the chitinases most similar to *St*Chi40, which suggests that their presence is significant. The disulfides could affect the thermodynamics of *St*Chi40 to enhance its thermostability and renaturation properties. Cystine residues should decrease entropy of the protein’s unfolded state, because once they are formed, the breaking of the covalent bond is energetically demanding and would require temperatures higher than ambient even for thermophiles.

Salt bridges are considered to be the stabilizing features in thermostable proteins. A comparison of the salt bridges in *St*Chi40 and its closest homologues shows that in this respect *St*Chi40 does not follow the trend (Table 1). It goes to show that thermostability in proteins is achieved by a mix of factors in various proportions and that, considered individually, they can be poor predictors of protein’s properties. In the case of *St*Chi40, counting salt bridges to assess its thermostability would be misleading.

Two sites in *St*Chi40 which are occupied by positively charged residues in all mesophilic and psychrophilic chitinases used in the comparison are substituted by Pro residues. The only exception is the thermophilic chitinase from *R. miehei*, where these sites are occupied by hydrophobic residues. The presence of Pro could serve to stiffen the local structure. Proline stands out among amino acids due to its restricted conformational possibilities, which should lower entropy of the protein’s unfolded state [34]. In this context, one should keep in mind that stability of a protein’s structure can be achieved not only through stabilizing the folded state, but also by “destabilizing” the unfolded state [35,36,37].

To summarize, a comparison of this thermostable protein with its cold-adapted homologue from *Arthrobacter* TAD20 reveals features that could be relevant to its thermostability: in particular, its compact structure with trimmed surface loops, disulfide bridges, one of which is additionally stabilized by S–π interactions due to close contacts of the cystine group with phenylalanine rings, and some unique proline residues that could promote shifting the equilibrium from the unfolded to the folded state. The respective contributions of these features to the stability of the protein are yet to be determined.

## 4. Materials and Methods

### 4.1. Biological Source

The chitinase under study comes from the *Streptomyces thermoviolaceus* bacterial strain (ATCC 15381). All enzymes used in the cloning procedures were from Takara and New England Biolabs. The TA cloning system was from InVitrogen (Watham, MA, USA). The column chromatography media were from Pharmacia (Pfizer, New York, NY, USA) or Clontech (Takara, Kyoto, Japan) and all the other chemicals were from Sigma-Aldrich (St. Louis, MO, USA) or Merck (Kenilworth, NJ, USA), in the highest analytical grade. Synthetic oligonucleotides were prepared by MWG (Munich, Germany). The *Streptomyces thermoviolaceus* bacterial strain was grown at 50 °C in a medium containing 10 g/L yeast extract (DIFCO), 5 g/L proteose peptone (DIFCO), 1 g/L K_2_HPO_4_, 0.2 g/L MgSO_4_.7H_2_0 (pH 7.2).

### 4.2. Plasmid and DNA Manipulations

Chitinase gene *chi*40 from *S. thermoviolaceus*, originally isolated as described by Tsujibo et al. [9,38], was cloned under the T7 promoter. The natural secretion signal sequence of *chi*40 was replaced by the pelB secretion sequence for efficient expression and export of *St*Chi40 to the periplasm and ultimately to the growth medium, as described by Christodoulou et al. [39]. PelB is a 22 amino-acid N-terminal leader sequence of pectate lyase B of *Erwinia carotovora* [40], which upon attachment to a protein directs it to the periplasm and out of the cell, while the signal peptide is removed by a signal peptidase. The protein was released from periplasm by osmotic shock tuned to disrupt only the outer membrane of the cells [41]. After pelleting the cell debris, the supernatant was adjusted to 1M ammonium sulfate and 20 mM Na-phosphate at pH 8.0 and applied on a Phenyl Sepharose column for hydrophobic interaction chromatography. The protein was eluted by a descending 1–0 M ammonium sulfate gradient. The purified and concentrated *St*Chi40 was finally purified to homogeneity by size exclusion chromatography to remove small molecular weight minor impurities and aggregates. The *St*Chi40 concentration was determined using a UV spectrophotometer at 280 nm. The molar absorption coefficient was calculated to amount to 104,195 M^−1^ cm^−1^ at 280 nm and used to determine the protein concentration. Mass spectroscopy analysis confirmed the calculated molecular weight and dynamic light scattering measurements indicated that purified *St*Chi40 was monomeric in solution (data not shown).

### 4.3. Crystallization and X-Ray Data Collection

After many crystallization attempts using the vapor diffusion method, only one crystal of *St*Chi40 grew after approximately 18 months. The well solution initially contained 1.9 M sodium malonate at pH 5.0. Crystallization drops were prepared by mixing the 30 mg/mL protein solution with the well solution in the 1:1 ratio. Further crystallization attempts involving the above conditions as well as various *St*Chi40 N-terminal mutants at different concentrations, adding the reaction products and substrates, changing the crystallization temperature (4 °C, 15 °C, 19 °C, 30 °C), and using other crystallization screens yielded no positive results.

X-ray diffraction data were collected on beam line I911-3 at the MAX-lab synchrotron in Lund using a Marresearch MX-225 detector. It was possible to collect three separate data sets from different parts of the single available crystal. Most images contained a large number of overlapping reflections causing severe problems with data processing. Close inspection of the frames and processing with various parameters allowed the best data set to be selected. The chosen data set consisted of 320 images, of which a continuous subset of images giving reasonable merging statistics, 71–240, was selected. The data were integrated and scaled with XDS [42]. A summary of the X-ray data collection and processing of the best data set is given in Table 2.

### 4.4. Structure Determination and Refinement

The phase problem was solved by molecular replacement using Phaser [43]. The search model was generated by the GeneSilico MetaServer, using various methods for protein prediction mainly based on homology modelling [44]. Two protein molecules in the asymmetric unit were identified with Phaser, indicating a solvent content of nearly 70%. The model was refined using the phenix.refine software [45]. TLS parameters [46] were refined for both protein molecules: four and five groups, accordingly, and restrained in refinement. The atoms related by non-crystallographic symmetry were restrained automatically in refinement. The final statistics are listed in Table 2. The atomic coordinates and structure factors have been deposited in the Protein Data Bank under accession code 4w5u.

## Figures and Tables

**Table 1:** Number of salt bridges and a comparison of residues in related chitinases.

Protein Name and Origin	Temperature Regimen	PDB Code	No. of Salt Bridges	*St*Chi40 Residues Discussed in the Text and the Residues that Substitute Them in the Related Proteins			
*St*Chi40 from *Streptomyces thermoviolaceus*	thermophilic	4w5u	5	His167	Glu214	Pro155	Pro264
Chitinase from fungus *Rhizomucor miehei*	thermophilic	5xwq	2	Lys170	Thr218	Phe158	Ala268
Chitinase A1 from *Bacillus circulans*	mesophilic	1itx	12	Arg192	Tyr246	Arg180	His291
Chitinase CrChi1 from *Clonostachys rosea*	mesophilic	3g6l	8	Lys162	Phe209	Arg150	His254
ChiA from *Serratia marcescens*	mesophilic	1ctn	13	Gln302	Tyr357	Arg290	His403
Chitinase B from fungus *Aspergillus niger*	mesophilic	6igy	10	Ala137	Phe184	Arg125	His229
Chitinase-h from insect *Ostrinia furnacalis*	mesophilic	5gpr	13	Gln295	Tyr350	Arg283	His396
ChiA74 from *Bacillus thuringiensis*	mesophilic	6bt9	10	Arg199	Tyr253	Arg187	His298
Chitinase from *Vibrio harveyi*	mesophilic	3b8s	9	Lys302	Tyr358	Arg290	His403
Chitinase B from *Arthrobacter* TAD20	psychrophilic	1kfw	13	Lys165	Tyr236	Arg152	His284

**Table 2:** Summary of X-ray data collection and refinement.

Space group	P6_1_22
Unit cell parameters	
*a = b* (Å)	183.2
*c* (Å)	130.8
Beamline	MAX-lab I911-3
Wavelength (Å)	1.0000
Resolution (Å)	49–2.77 (2.94–2.77) *
R_merge_^#^	0.15 (1.01)
Completeness (%)	99.4 (98.1)
Observed reflections	333,347
Unique reflections	33,240
<I/σ(I)>	13.5 (1.9)
Multiplicity	10.0
R ^§^/R_free_^$^	0.15/0.20
Protein atoms	5572
Ligand atoms	14
Water molecules	74
Average B factor (Å^2^)	62
Error in the Luzzati plot (Å)	0.15
R.m.s. deviation from ideal	
bond lengths (Å)	0.017
bond angles (°)	1.63
Ramachandran plot (%)	
favored	96
allowed	4
outliers	0
PDB code	4w5u

* Values in parentheses are for the highest resolution shell. ^#^ R_merge_ = Σ_hkl_Σ_i_|I_i_(hkl) − <I(hkl)>|/Σ_hkl_Σ_i_I_i_(hkl), where I_i_(hkl) is the integrated intensity of a given reflection and <I(hkl)> is the mean intensity of multiple corresponding symmetry-related reflections. ^§^ R = Σ_hkl_||F_obs_| − |F_calc_||/Σ_hkl_ |F_obs_|, where F_obs_ and F_calc_ are the observed and calculated structure factors, respectively. ^$^ R_free_ is R calculated using randomly chosen reflections excluded from the refinement.

## Abbreviations

*St*Chi40	Chitinase 40 from *Streptomyces thermoviolaceus*
GHJ	Glycoside hydrolase
ChBD	Chitin-binding domain
CBMs	Carbohydrate-binding modules
r.m.s.	Root-mean-square
TIM	Triose-phosphate isomerase
EC	Enzyme Commission (number)
ChiA	Chitinase A
ChiB	Chitinase B
NAG	N-Acetylglucosamine
PDB	Protein Data Bank
TLS	Translation/Libration/Screw
ATCC	American Type Culture Collection
